# Decreased relative risk of pneumococcal pneumonia during the last decade, a nested case-control study

**DOI:** 10.1186/s41479-018-0053-6

**Published:** 2018-09-25

**Authors:** Carlos M. Luna, Laura Pulido, Michael S. Niederman, Alberto Casey, Diego Burgos, Sebastián D. Leiva Agüero, Alejandra Grosso, Evangelina Membriani, Andrea C. Entrocassi, Marcelo Rodríquez Fermepin, Carlos A. Vay, Susana Garcia, Angela Famiglietti

**Affiliations:** 1 Department of Medicine, Pulmonary Diseases Division; Hospital de Clínicas, Universidad de Buenos Aires, Ayacucho, 1850, piso 5 dto B, 1112 Buenos Aires, Argentina; 2 Department of Chemical Biochemistry, Immuno-Serology Section, Faculty of Pharmacy and Biochemistry; Hospital de Clínicas, Universidad de Buenos Aires, Buenos Aires, Argentina; 3 Department of Chemical Biochemistry, Section Microbiology, Faculty of Pharmacy and Biochemistry; Hospital de Clínicas, Universidad de Buenos Aires, Buenos Aires, Argentina; 4 Pulmonary and Critical Care, New York Presbyterian/ Weill Cornell Medical Center, New York, USA

**Keywords:** Community acquired pneumonia, *Streptococcus pneumoniae*, Urinary antigen test, Prevalence, Pneumococcal conjugate vaccine

## Abstract

**Background:**

*Streptococcus pneumoniae* (SP) is one of the most common pathogens of Community-Acquired Pneumonia (CAP), but recent reports suggest that its incidence may be declining in relation to the use of the conjugate 13-valent pneumococcal vaccine in children. We compared the result of the immunochromatographic SP urinary antigen test (SPUAT) and clinical outcomes in patients with CAP admitted in two periods of time: 2001–2002(CAP1) and 2015–2016(CAP2).

**Methods:**

This was a matched nested case-control study of two prospectively recorded cohorts of patients admitted with CAP, with SPUAT and blood culture performed in all patients. CAP2 cases and CAP1 controls were matched for age ± 4 years, sex, and Pneumonia Severity Index (PSI) score ± 10 points. Odds ratios (OR) for having SPUAT positive was estimated by conditional logistic regression. A multivariate model assessed the contribution of individual variables.

**Results:**

Four hundred ninety-eight patients were recruited; 307 during the CAP1 and 191 during the CAP2 periods. Comparing both periods we observed differences, in age, PSI score, and the percentage of smokers, outpatients, previously immunized with pneumococcal vaccine, and positive SPUAT. On the other hand, mortality, admission from nursing homes, pneumococcal bacteremia and hospital admission were not different. After matching, pneumonia due to SP per the SPUAT was observed in 34(23.4%) of CAP1 and in 12(8.3%) of CAP2 patients (*p* < 0.001), and 6/145 CAP1 vs 33/145 CAP2 patients had received pneumococcal immunization before their admission (*p* < 0.001). A multivariate analysis confirmed that, independent of falling into PSI class 5, having not received the pneumococcal vaccine and having not survived the episode of pneumonia, there were two factors that increased the probability of having SPUAT positive: developing pneumonia during the CAP1 period (OR = 1.23) and having pneumococcal bacteremia (OR = 2.66).

**Conclusion:**

We observed a reduction of the role of SP as pathogen, along with an increase in the number of patients who received pneumococcal immunization before admission, in 2015-2016 compared to 2001-2002. In addition, the use of conjugate 13-valent vaccine, starting in 2012 for childhood immunization, could be an additional factor contributing to these changes, as a result of early herd immunity in adults pneumonia.

## Background

Community acquired pneumonia (CAP) is one of the most common causes of morbidity and mortality worldwide [1] and *Streptococcus pneumoniae* (SP) has been found to be its most common pathogen [2]. The frecuency of pneumococcal pneumonia might be decreasing following widespread use of the 13-valent pneumococcal vaccine in children, and in people 65 years of age or older [3].

Disease caused by SP is a major public health problem worldwide, leading to invasive CAP, meningitis, and bacteremia. Non-invasive illnesses, such as non-bacteremic pneumonia, otitis media, bronchitis and upper airway infections are common but less severe [4]. Besides its role as a pathogen, SP is asymptomatically carried in the nasopharynx by up to 50% of infants and up to 5% of adults [5]. Colonization occurs before disease, and transmission is from child to child and from children to adults can occur [6]. In addition to the traditional microbiological diagnosis of pneumonia based on respiratory specimens and blood culture, the use of immunochromatographic SP urinary antigen test (SPUAT) in patients with CAP is also recommended for diagnosis by international guidelines [7]. Sensitivity and specificity of SPUAT in adults for the etiologic diagnosis of pneumococcal CAP have been found to be 57.9–75% and 96.6–99.7%, respectively [8–11]. Antibodies to capsular polysaccharide antigen provide serotype-specific protection against serious infection, and the pneumococcal vaccines are designed to cover the serotypes most commonly associated with severe pneumococcal disease [12]. Vaccination has greatly reduced the burden of pneumococcal disease.Two types of pneumococcal vaccines are available in Argentina (country where this study was performed): the 23-valent pneumococcal polysaccharide (non-conjugate), (PPV23) and the 13-valent polysaccharide conjugate vaccine (PCV13).

By 2016, the national committee on immunizations in Argentina (CoNain) recommended the PPV23 for all individuals ≥65 years of age and for those younger, with comorbidities. Universal PCV13 vaccination for children was initiated in Argentina in 2012 [at 2, 4 and 6 months and 1 year of age (3 + 1 schedule)], with a coverage higher than 90% [13]. Results of the Community-Acquired Pneumonia immunization Trial in Adults (CAPiTA), a randomized controlled trial in elderly adults in the Netherlands, demonstrated the efficacy of PCV13 to prevent IPD and pneumonia caused by homologous serotypes [14]. A decline in the incidence of pneumococcal pneumonia in adults has been observed after PCV13 introduction in children, indicating an early indirect protection (so called herd effect) on adult disease, as a consequence of childhood vaccination [15]. These effects may accrue over the coming years with implications for national pneumococcal vaccination policies in adults.

## Objectives

To compare the SPUAT result (a sensitive and highly specific test to detect pneumococcal infection), the clinical findings and outcomes, in patients with CAP admitted during two different periods of time: 2001–2002 (CAP1) and 2015–2016 (CAP2), trying to define if there were any differences in the frequency and clinical features of pneumococcal CAP during these two periods.

## Methods

### Patients

The cases eligible for the study were adults over 17 years-old, with CAP (which met the inclusion criteria described below), consulting the Emergency Department or admitted to the wards of the Department of Medicine and the critical care areas of the Hospital de Clínicas, University of Buenos Aires, from January 1st 2015 to December 31st, 2016. The controls were patients admitted to the same institution from January 1st 2001 to December 31st, 2002. Cases and controls were evaluated in an identical fashion during the two periods of time. The criteria published in a National Clinical Practice Guideline were applied in decision making about the site of care to the patients who were admitted (Table 1) [16]. Inclusion criteria were: having a presumptive diagnosis of pneumonia within the first 24 h of admission to the study; presence of a new infiltrate on chest radiograph, and clinical findings confirmatory of at least one “major criterion” such as cough, sputum, or fever > 37.8C; or at least two “minor criteria”, including pleuritic chest pain, dyspnea, altered mental status, findings consistent with consolidationon physical exam, and leukocyte count > 12,000/ mm^3^ [17]; having had testing for the detection of SPUAT and blood cultures during their initial microbiological evaluation at admission. SPUAT was carried out from freshly obtained urine samples according to the manufacturer’s instructions (results read visually at 15 min and recorded as positive, with no interpretation of color intensity, or negative). All samples were stored at ≤ − 70 °C and assayed later, directly after thawing, Exclusion criteria included: having been hospitalized for any reason within the previous 14 days; no chest radiographic confirmation of a new infiltrate or attributing the abnormality only to a noninfectious etiology; a confirmed diagnosis of lung cancer before admission to the study; patients with a known diagnosis of HIV at the time of enrollment, or high doses of immunosuppressants (> 20 mg of prednisone or other corticosteroid dose equivalent) in chronic treatment (over 20 days).

**Table 1:** Criteria for hospital admission, Luna CM, et al. [16]

1) Presence of two or more of the following: age > 65 years; chronic pulmonary disease; congestive heart failure; chronic kidney failure; chronic liver disease; malignant neoplasm; diabetes mellitus; hospitalization for pneumonia during the past year. 2) Respiratory rate ≥ 30 / minute. 3) Low blood pressure: systolic < 90 and/or diastolic: < 60 mmHg). 4) Poor respiratory mechanics. (paradoxical respiration. Circulation. etc.). 5) Any of the following: PaO_2_ < 60 mmHg; SaO_2_ < 89%; PaCO2 > 50 mmHg with acidosis; white blood cell count < 4000 or > 30.000/mm^3^; BUN > 25 mg/dl; hematocrit < 30%. 6) Alteration of consciousness. 7) Serious swallowing disorder suggesting likely aspiration. 8) Suppurative complications (pericarditis. Arthritis. empyema. Etc.) 9) Severe chest radiograph involvement (multilobar, bilateral, cavitation, progression > 50% over a previous RX) 10) Reasons suggesting inaccurate treatment compliance.	

Mortality: was defined as death that occurred within 30 days of the diagnosis of CAP.

### Evaluation

Clinical evaluation included: risk factors such as smoking and comorbidities (chronic lung disease, diabetes; chronic heart, renal or liver disease and history of malignancy). Findings on the clinical exam were recorded for each patient. Pneumonia severity index (PSI) [18] was calculated .

Radiological assessment: the radiological patterns considered to support the diagnosis of pneumonia included the presence of: focal or multi-focal infiltrates, airspace infiltrates or an interstitial or miliary pattern. Pleural effusion without any parenchymal radiological findings was not included.

Microbiologic evaluation: urine samples collected in the emergency department or on the ward in the first 24 h to test for SPUAT (BinaxNOW ®, Portland, ME, and Alere Inc., Waltham, MA, USA) in accordance with the manufacturer’s instructions. Blood cultures were drawn in all patients at admission. Culture of respiratory specimens for common aerobic bacteria were performed in a number of cases, but not systematically in all patients.

Blood analyses: all the hospitalized and some ambulatory patients entered into the study had blood drawn for white blood cell count and routine biologic analyses.

Demographic data (age and sex), the PSI score, site of care (ambulatory or admitted to the general ward or the ICU), presence of criteria for receiving pneumococcal vaccine, results of the blood culture, admission from a nursing home, past medical history including presence of comorbidities, smoking history and vaccination against SP were recorded.

### Therapy and follow-up

Patients admitted to the study were treated per the criteria adopted by the attending physician and followed for up to 30 days. In brief, all patients received empiric antimicrobial therapy as early as possible and concordant with the National Guidelines, and fluid resuscitation with intravenous crystalloids for patients with hypotension.

### Data analysis

We carried out a nested case-control, cohort study; cases (the CAP2 patients) and controls (the CAP1 patients) were prospectively enrolled during the two periods of study. Cases were matched to controls for age ± 4 years, sex, and PSI score ± 10 points, allowing for the possibility of finding different etiologies and frequencies of pathogens. Results are expressed as mean and standard deviation for age, or as number and percentages for categorical variables. Age was assessed by the *t- test* and categorical variables were assessed by the chi square or Fisher exact test. The relative risk (RR) and 95% confidence interval (95%CI) of the cathegorical variables and their change over the time periods studied, were analyzed. A multivariate analysis, using a conditional logistic regression model, suggested for matched case-control studies [19], with the dependent variable being the positivity of the SPUAT, was performed. Confounding factors including PSI class 5, having not received the pneumococcal vaccine and having not survived the episode of pneumonia, were used for adjustment to calculate the odds ratio (OR) and 95%CI. All data management and statistical analyses were performed using the SPSS 21 processor (SPSS Inc., Chicago, IL).

This study was approved by the ethics committee of the hospital, but patient-informed consent was considered unnecessary because of the observational nature of the study, but the study team explained the potential risks related to drawing blood, including the nature of potential risk of blood drawing.

## Results

### Clinical characteristics of the cohort

**Fig. 1:**
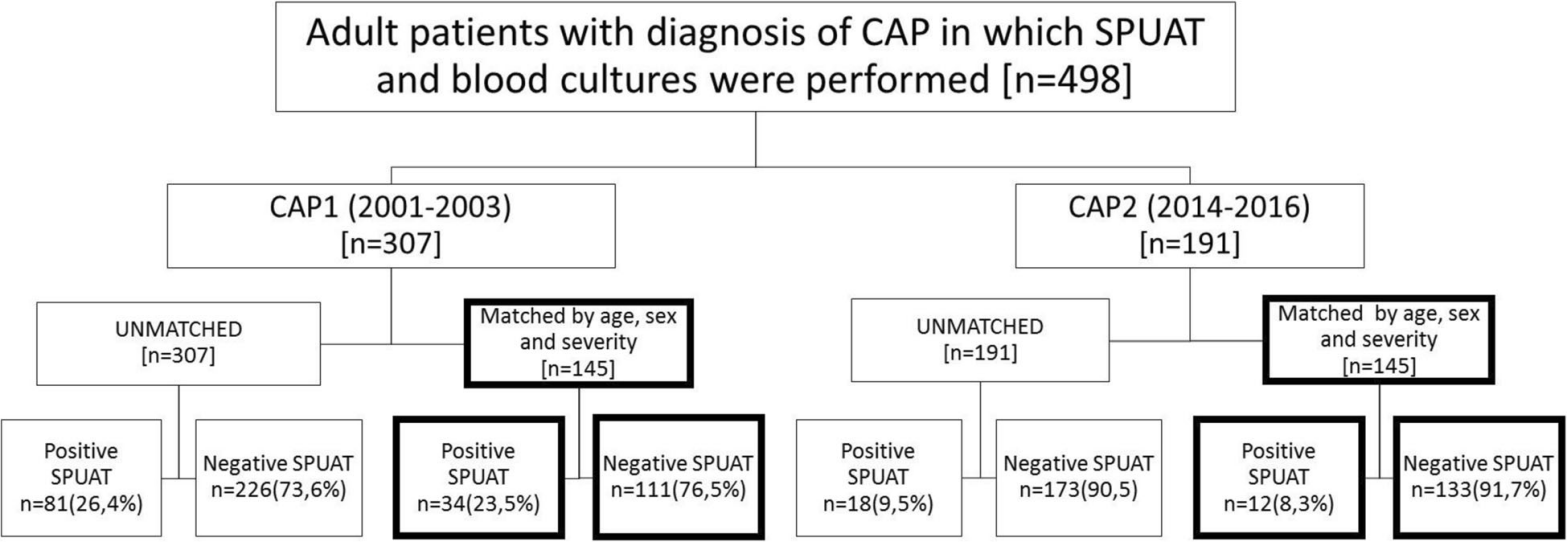
Distribution of the patients in the cohorts CAP1 and CAP2 in general and considering the approaches analyzing both cohorts, unmatched and matched. The unmatched patients, both from CAP1 and CAP2 cohorts, were excluded because they had no pair with comparable age, sex and PSI score. The number of patients excluded from the case-control analysis were 162 from the CAP1 and 46 from the CAP2 cohorts

A total of 498 patients with a clinical diagnosis of CAP made on either an outpatient basis or during the first 24 h of hospitalization were consecutively recruited. In all patients, at least one specimen for SPUAT and two sets of blood cultures were collected during their initial microbiological evaluation. Among these patients, 307 were recruited during the CAP1 period and 191 during the CAP2 period, respectively (Fig. 1).

Some significant differences were observed comparing the characteristics of the patients (unmatched) recruited during the two different periods, CAP1 and CAP2, including: age, 64.6 ± 21.2 vs. 70.3 ± 19.1 years old (*p* = 0.002); PSI score, 110.7 ± 39.8 vs 93.8 ± 41.1 (*p* < 0.001); and percentages of smokers, 9.1% vs.17.3% (*p* = 0.007). The rates of hospitalization for each age group were 75.5% and 61.7%, for CAP1 and for CAP2, respectively (*p* = 0.001) (Table 2, panel A). Also, we found significant differences among the percentage of patients who had received pneumococcal immunization, 4.2% vs 18.8% (*p* = 0.001) and the positivity of the SPUAT, 25.0% vs 9.5% (*p* = 0.001) respectively, comparing CAP1 and CAP2.

**Table 2:** Comparison of demographic and clinical characteristics among patients with CAP during the CAP1 and the CAP2 periods unmatched and matched (case control). Figures are means (±SD) and n (%). The *p* value and risk ratio (RR)

	PANEL A (UNMATCHED)			PANEL B (MATCHED)			
Variable	CAP1 (*n* = 307)	CAP2 (*n* = 191)	p value	CAP1 (*n* = 145)	CAP2 (*n* = 145)	p value	RR
Age, year (±SD)	64.6 ± 21.2	70.3 ± 19.1	0.002	67.8 ± 20.4	67.7 ± 20.4	0.484	
Male Sex	160 (58.6%)	93 (48.7%)	0.749	74 (51.0)	74 (51.0)	1.000	1.000
PSI class 5	106 (34.5%)	43 (22.5%)	< 0.001	37 (25.5%)	33 (22.8%)	0.575	1.077
Admitted from nursing home	43 (14.0%)	27 (14.1%)	0.972	12 (8.3%)	23 (15.9%)	0.082	0.657
Managed as outpatients	75 (24.4%)	73 (38.2%)	0.003	51 (35.12%	54 (37.2%)	0.706	0.956
Admitted to the hospital	232 (75.5%)	118 (61.7%)	< 0.001	94 (64.8%)	91 (62.8%)	0.716	0.968
Admitted (general ward)	176 (57.3%)	100 (52.3%)	0.402	83 (57.2%	77 (53.1%)	0.481	1.088
Admitted (ICU)	56 (18.2%)	18 (9.4%)	0.007	11 (7.6%)	14 (9.7%)	0.552	0.870
Positive blood culture	12 (3.9%)	7 (3.7%)	0.954	7 (4.8%)	6 (4.1%)	0.784	0.911
Requiring mechanical ventilation	16 (5.2%)	6 (3.1%)	0.012	4 (2.8%)	5 (3.4%)	0.748	0.886
Pneumococcal immunization	13 (4.2%)	36 (18.8%)	< 0.001	6 (4.1%)	33 (22.7%)	< 0.001	0.255
Pneumococcal. vaccine Indication	247 (80.4%)	142 (74.3%)	0.108	109 (75.2%)	103 (71.0%)	0.438	1.114
Positive SPUAT	81 (26.4%)	18 (9.5%)	< 0.001	34 (23.4%)	12 (8.3%)	< 0.001	2819
Current smoker	28 (9.1%)	33 (17.3%)	0.007	15 (10.3%)	20 (13.8%)	0.396	0.841
Chronic lung disease	91 (29.6%)	41 (21.5%)	0.041	37 (25.3%)	28 (19.3%)	0.184	1.186
Cardiac disease	68 (22.2%)	38 (19.9%)	0.558	21 (14.5%)	29 (20.1%)	0.243	0.813
Diabetes	29 (9.5%)	22 (11.5%)	0.464	10 (6.9%	13 (8.9%)	0.706	0.538
Hepatic disease	6 (1.9%)	6 (3.1%)	0.412	2 (1.4%)	3 (2.1%)	0.681	0.797
Renal failure	30 (10.8%)	19 (9.9%)	0.951	10 (6.9%)	5 (3.4%)	0.112	1.358
Descesed	64 (20.8%)	30 (15.5%)	0.143	23 (15.9%)	22 (15.2%)	0.870	1.026

Considering the indications for pneumococcal vaccination according to the national recommendations in Argentina (including age > 65 years, comorbidities, splenectomy, and cerebrospinal fluid leakage); 247 (80.4%) patients had an indication for pneumococcal vaccination in the CAP1 and 142 (74.2%) patients, in the CAP2 time period.

### Nested case-control study

It was possible to successfully pair 145 CAP2 patients, age- (± 4 years), sex- and severity- (PSI score ± 5 points) matched, with 145 CAP1 control subjects. Forty-six cases were excluded because it was not possible to find a control among the CAP1 patients for them. The result of the comparison of CAP1 with CAP2 matched patients’ findings in this nested case-control study is displayed in Table 2, panel B.

Comparing CAP2 with CAP1 matched patients, most of the variables were not different. The exceptions were the increasing rate of pneumococcal immunization, 4.1% versus 22.7%, (RR = 0.2549, 95% confidence interval [CI] {0.1205 to 0.5390} 0.), *p* < 0.001 and the declining rate of positive SPUAT, 23.4% vs. 8.3%, (RR = 1.6248, 95% CI [1.3041 to 2.0243]), *p* < 0.001, comparing the two periods (Table 2). This last finding represents a reduction in the RR of pneumococcal pneumonia that happened in the CAP2 period, compared with the CAP1 period.

Pneumococcal vaccination had been performed using the PPV23 vaccine in all cases during the CAP1 period. The same happened with the majority of those included in the CAP2 period, as PCV13 vaccine was approved for use in adults in 2012, and only indicated in adults older than 65 years-old by the CoNain in 2016, after the CAPiTA study was published. The CAPITA study demonstrated among this population that PCV13 was effective in preventing vaccine-type pneumococcal, bacteremic, and nonbacteremic CAP and vaccine type invasive pneumococcal disease, but not in preventing community-acquired pneumonia from any cause [14, 20].

Interestingly, although the positivity of the SPUAT was significantly different, showing a reduction in the RR of SP as a pathogen of pneumonia, the rate of bacteremia due to SP did not change during this time. All SP isolated in blood cultures were susceptible to penicillin (Clinical and Laboratory Standards Institute MIC breakpoints for patients without meningitis treated with intravenous penicillin were < 2 μg/mL).

Mortality was also not different comparing CAP1 with CAP2 periods, in the matched population.

The result of the univariate and multivariate analyses using the positivity of the SPUAT as the dependent variable, the variables evaluated and their OR, the 95% CI, and the *p* values are displayed in Tables 3 and 4. These results confirm that, independent of the presence of a PSI risk class 5, of having a history of receving pneumococcal vaccine and of not surviving the episode of pneumonia, there were two factors that increased the chance of having the SPUAT positive: developing pneumonia during the CAP1 period in 2001–2002 (with an OR of 1.23) and having pneumococcal bacteremia (with an OR of 2.66).

**Table 3:** Conditional Logistic Regression model for matched cases and controls, Univariate Analysis, The odds ratio (OR), the 95% confidence index (CI 95%) and the *p* value are displayed

Variable	OR	CI 95%	*p* value
CAP1 versus CAP2 Períod	1.23	0.30 to 1.80	0.006
Sex	0.62	−0.22 to 1.47	0.148
Nursing Home	−1.93	−4.00 to 0.14	0.067
PSI = 5	0.88	−0.01 to 1.77	0.053
CURB 65	0.26	− 0.11 to 0.64	0.168
Positive blood cultures	2.80	1.26 to 4.35	< 0.001
Positive sputum	2.44	0.20 to 4.67	0.033
Admitted to general ward	1.63	0.60 to 2.66	0.002
Outpatients	−1.85	−3.03 to −0.68	0.002
Admitted to the ICU	−0.19	−1.60 to 1.23	0.800
Decesed	0.89	−0.20 to 1.99	0.110
Pneumococcal vaccination	−0.25	−1.46 to 0.95	0.680

**Table 4:** Conditional Logistic Regression model for matched cases and controls, Multivariate Analysis, showing that regardless of PSI class = 5, having received pneumococcal vaccine, and age (in addition to all the variables by which the individuals were matched), the only two factors that increased the chance of having positive SPUAT were belonging to the CAP1 (OR 2.66) cohort and having positive blood cultures (OR 2.66)

Variable	OR	CI 95%	*p* value
CAP1 versus CAP2 Períod	1.23	0.30 to 2.17	0.01
Positive blood cultures	2.66	0.80 to 4.52	0.005
PSI = 5	0.005	−0.013 to 0.023	0.590
Decesed	−0.19	−1.57 to 1.20	0.790
Pneumococcal vaccination	0.27	−1.2 to 1.73	0.720

## Discussion

The most important findings of our study are: (1) the relative risk of pneumococcal pneumonia, as per SPUAT positivity in a cohort of patients treated for CAP in a University Hospital (Hospital de Clínicas, University of Buenos Aires), during a 2 year (CAP2), was reduced overall to a ratio of 0.34 (*p* < 0.001), compared with a control cohort, treated 14 years earlier (CAP1), matched by age, sex and severity of illness; (2) the f history of pneumococcal vaccination (mainly PPV23) among the CAP2 group of patients increased overall to a ratio of 0.55 (*p* < 0.001), even though the percentage of patients indicated to receive the vaccination did not change. In addition, rates of pneumococcal bacteremia and overall mortality did not change over the two time periods. Reported mortality was comparable to the rates observed in the UK (Chalmers JD, et al...) and US (Morril HJ, et al) [21, 22]. Out matched, nested case-control methods run the risk of over-matching, and thus we also analyzed the positivity of the SPUAT observed in this study using a multivariate method. This analysis was based on the linear logistic model (conditional logistic model), recomended for the analysis of case-control studies with pairwise matching. This technique enabled us to investigate the effect of several variables simultaneously in the analysis, while allowing for the matched design. This multivariate analysis demonstrated that only two factors increased the chance of having a positive positive SPUAT: developing pneumonia in the CAP 1 period (OR = 1.23) and having pneumococcal bacteremia (OR = 2.66). The latter was expected, but confirms the robustness of our analysis.

The SPUAT test, provides results in 15 min, detecting the C-polysaccharide cell wall antigen common to all SP strains [23]. The sensitivity and specificity of the SPUAT have been evaluated and most studies demonstrated high specificity near 100% and moderate to high sensitivity. Horita et al., found in a meta-analysis, a pooled specificity of 0.75 and a pooled sensitivity was 0.95. This high sensitivity and specificity make SPUAT a useful tool in clinical practice to predict negatively or positively the role of SP in the etiology of CAP. Ortega et al. found evidence that links the degree of the SPUAT positivity to the severity of the pneumonia [24]. Some studies published between 2001 and 2007 in patients with a clinical diagnosis of CAP showed that SPUAT was positive in a range between 16 and 33% of patients [25–28].

The introduction of PCV7 into the infant immunization programs in developed countries beginning in 2006, was highly effective in reducing the incidence of invasive and non-invasive pneumococcal disease. Rodrigo et al., beginning in 2008, conducted a 5-year prospective cohort study of adults admitted to hospital with CAP in Nottingham, UK [15]; they observed a reduction in the incidence of the number of adult patients hospitalized for pneumococcal CAP [incidence rate ratio (IRR) per year 0.84, 95% CI 0.80–0.89; *p* < 0.001] over the 5-year period of the study. The incidence of CAP due to serotypes included in the PCV7 declined by 88% (IRR 0.12, 95% CI 0.08–0.20; *p* < 0.001), and CAP due to the additional 6 serotypes in PCV13 declined by 30% (IRR 0.70, 95% CI 0.51–0.96; *p* = 0.024). This study and other previous studies described the finding of so-called herd immunity, reducing the incidence of pneumococcal pneumonia in children and adults, after the introduction of PCV7 and PCV13 into the infant immunization programs [29, 30]. Other authors found that although PCV13 protects against key serotypes that increased after routine immunization with the PCV7, its potential for herd immunity and serotype replacement is uncertain [31–37]. In Argentina, the percentages of coverages for the 1st and 3rd doses of PCV13 have been appropriate and comparable with the coverage observed in developed countries such as the United States, after its introduction in 2012. Our study suggested a potential benefit to adults, of the immunization with pneumococcal vaccine in children, leading to a dramatic decline in non-invasive pneumooccal CAP, diagnosed by SPUAT, but no change in bacteremic pneumococcal CAP.

This study has several limitations, including: the diagnosis of pneumococcal pneumonia was based on the positivity of the SPAUT, a methodology that highly specific, but can produce between a 25% and 42.1% rate of false negatives [8–11]. Blood cultures were performed to all patients, but respiratory cultures and/or serotyping were not available for all patients. Also, we could not analyze the effect of concordant therapy, because this was not recorded in all patients in the CAP1 cohort.

Among the strengths of our study, is our comparison of two cohorts, one treated after the implementation of PCV vaccination in children in the calendar in 2012, with another comparable cohort treated before the implementation of this pneumococcal vaccine, allowing us to compare the frequency of pneumococcal pneumonia, including by multivariate analysis.

## Conclusions

The result of this comparison demonstrated that the RR of pneumococcal pneumonia, diagnosed as per SPUAT positivity, was reduced in more recent years, leading us to speculate about possible explanations. The frequency of SP as the causative microorganism of CAP was reduced to a ratio of 0.34, compared with the control cohort, 23.4% vs 8.3% (*p* < 0.001), although there was no change in the frequency of bacteremic pneumococcal pneumonia. The introduction of pneumococcal vaccine might account for a decline in pneumococcal pneumonia by either inducing immunity in immunized at risk patients or by its herd immunity impact of childhood vaccination on the community, but this could be confirmed only in studies demonstrating a reduction of the serotypes represented by the vaccine.

## Abbreviations

CAP: community-acquired pneumonia.
CAP1: 2001–2002 period of time
CAP2: 2015–2016 period of time
CAPiTA: Community-Acquired Pneumonia immunization Trial in Adults
CoNain: the national committee on immunizations in Argentina.
OR: odds ratio
PCV13: the 13-valent pneumococcal conjugate vaccine.
PPV23: the 23-valent pneumococcal polysaccharide (non-conjugate) vaccne.
PSI: Pneumonia severity index
RR: relative risk
SP: *Sterptococcus pneumoniae*
SPUAT: immunochromatographic *Streptococcus pneumoniae* urinary antigen test
